# The recalescence rate of cooling curve for undercooled solidification

**DOI:** 10.1038/s41598-019-56079-6

**Published:** 2020-01-28

**Authors:** Junfeng Xu, Tian Yang, Zhuo Li, Xiao Wang, Ying Xiao, Zengyun Jian

**Affiliations:** The Shaanxi Key Laboratory of Photoelectric Functional Materials and Devices, Xi’an Technological University, Xi’an, Shaanxi 710021 P.R. China

**Keywords:** Materials science, Physics

## Abstract

Recalescence rate (*R*) in cooling curve is well known that affected by undercooling in solidification, but the accurate relationship of them is not clear yet. In this paper, based on the undercooled solidification of Fe-B alloy, the factor affected on recalescence process was investigated. The relationship *R* = *V*Δ*T/D* was first found, where *V* is the growth velocity, Δ*T* the recalescence degree (approximate the undercooling), *D* the focus region diameter dependent on the distance of the pyrometer. With this result the solidification interface growth velocity can be predicted from recalescence of cooling curve, vice versa. In addition, an approximate relation between growth velocity and the size of the critical nucleus was shown.

## Introduction

The liquid states, such as water drops and metallic melts, can be maintained at temperatures which are many degrees below their true freezing points, i.e. undercooled state^1^. Under undercooled state, temperature will rise suddenly due to the fusion latent heat release, i.e. recalescence. Recalescence is discovered and given the generic title’recalescent’ early in 1868^2^. At that time, the thermoelectric pyrometer was unknown, thus the recalescence curves could not be record although it can be observed. In 1898, Roberts-Austen W.C. revealed that the recalescences had been found in metallic gold, copper, bismuth, antimony, lead, and tin, has been described it as “A sudden glowing in a cooling metal caused by liberation of the latent heat of transformation”. Now then on, many studies pay operational attention on the recalescence of solidification process^3–15^. Olefield reported a furnace could measure the recalescence curve in casting. Flemings *et al*. measured the solidification rate in undercooled melts with a video system combining recalescence curve^4^. Suzuki *et al*. studied the recalescence in the solidification of Fe-P alloy, and they estimated the dendrite growth velocity with sample diameter divided by the recalescence times from thermal data. Although this method is not accurate owing to the recalescence time does not equals to solidification time actually, the experiment demonstrated that the recalescence rate have similar change tendency with that of the dendrite tip velocity^5^. Subsequently, Levi *et al*. revealed the connection of the recalescence curves and transition fraction, although the relationship between the transition fraction and transition time is still a problem^6^. Yahosseini *et al*. proposed a model to predict the recalescence process during solidification of metal droplets in the gas atomization process^7^. Shukla *et al*. also gave a mathematical model (including recalensece process) based on classical nucleation theory to predict undercooling of droplets^8^. Lee *et al*. developed the model for predict cooling curves and solid fraction for eutectic solidification of Gas-atomized alloy droplets^9^. All of them, however, cannot predict the recalescence (temperature change) actually because recalescence is also related to the parameters of thermometer (see section Discussion). By computer simulation, Geer *et al*. has given a numerical model for the prediction of grain size in inoculated castings on aluminum alloys. But the recalescence temperature change details was neglected due to unknown of the recalescence rate relationship^10^. Liang *et al*. studied the correlation of recalescence with grain refinement of magnesium alloys, and they found that grain size depends on the recalescence undercooling^11^. Bejan has reported the theoretically reason for the recalescence curve must be S-shaped in accord with the constructed law of design in nature^12^. Kapturkiewicz *et al*. gave the cooling curve model of austenite process although it was not fitting well with experiment^13^. Chen studied the eutectic modification level of Al–7Si alloy by computer aided recognition of thermal analysis cooling curves^14^. Marcano determined the fraction determination by using cooling curve analysis^15^. Li *et al*. employed High speed camera (HSC) to monitor the recalescence behaviors of single solid solution phase and eutectic in Al_2_O-ZrO_2_ system^16^. For all those studies, it is just known the recalescence rate increases with the undercooling, but it is not clear that what is the accurate relationship between recalescence rate and liquid-solid interface growth velocity. Without the relationship, the thermal history (cooling curves) and the growth velocity of solidification can not be predicted directly by each other.

In this study, the recalescence rate and growth velocity will be investigated by undercooled solidification of Fe-3.97 wt% B alloy melt. Then the simple relationship was further deduced and discussed.

## Results

**Figure 1:**
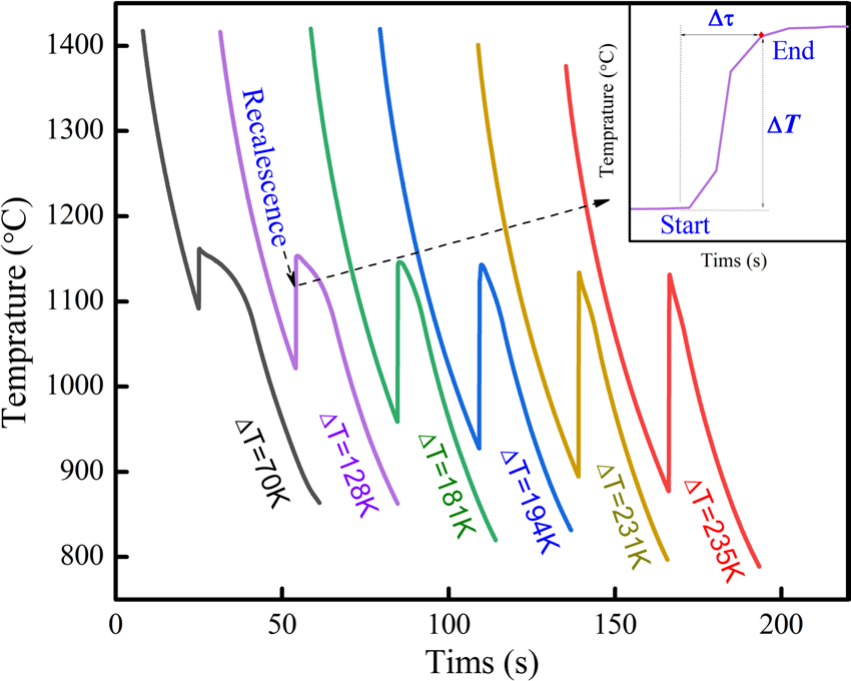
Cooling curves of hypoeutectic Fe-3.97 wt%B alloy.

If solidification processes at a certain undercooling, the recalescence will be observed in the cooling curves. Figure 1 shows the cooling curves of Fe-3.97 wt%B alloy solidified under different undercooling(L → Fe + Fe_2_B). As shown in the inset of Fig. 1, recalescence process display as ‘S-shape’ in transformation. Given the recalescence time as Δτ and recalescence temperature as Δ*T*, thus the recalescence rate R can be expressed as *R* = Δ*T*/Δτ. As can be seen, the recalescence process does not varies linear with time, thus the recalescence rate R is average value. Schleip el al have given the measurement method of Δτ in ref. ^17^. It is worth noting that the recalescence highest temperature for all the cooling curves is very close in resemblance to the eutectic melting point (1174 °C) in rapid solidification. This is because that the eutectic transition is similar to that of pure metal, i.e. solidification interface is uniform in temperature and composition.

**Figure 2:**
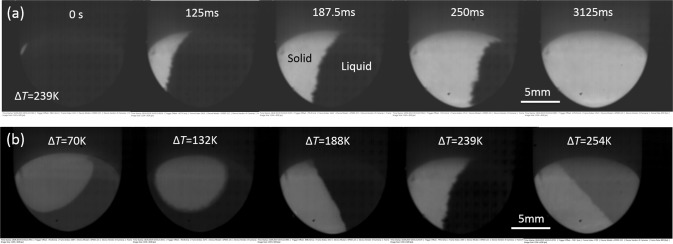
HSC images: (**a**) recalescence process of Δ*T* = 120 K; (**b**) solidification interface with different undercooling.

To observe the recalescence in sample surface, Fig. 2a shows the solidification interface moving process with undercooling of Δ*T* = 120 K from HSC. Figure 2b shows the solidification interfaces for difference undercooling’s. It is found that the morphologies of interfaces have minor differences for different undercoolings (Δ*T* = 70 K~254 K), but the moving rate changes rapidly. The growth velocity of solidification interface (*V*) are determined from interface moving distance (*S*) divided by the times (*t*), i.e. *V* = *S/t*.

**Figure 3:**
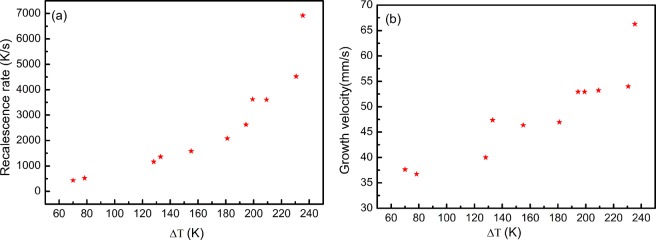
Recalescence rate from cooling curves (**a**) and solidification interface growth velocity from HSC images (**b**).

**Figure 4:**
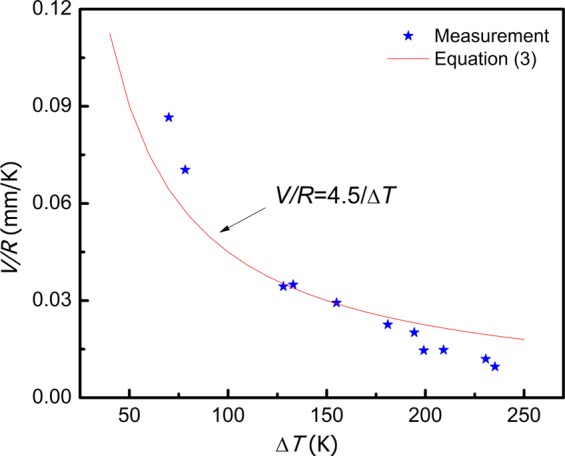
The ratio of recalescence rate and growth velocity.

Figure 3a shows the recalescence rate *R* measured from the cooling curve like Fig. 1. It can be seen that the value *R* increases exponentially with undercooling. Figure 3b shows the interface moving velocity *V* measured from the HSC image like Fig. 2a. As seen, the value of *V* also increases exponentially with undercooling. Thus, the values of *R* and *V* have a similar change trend. Therefore, there is connection between them. To study the relation of *V* and *R*, the value of *V/R* is shown in Fig. 4. It can be found the value of *V/R* is not a constant, but it decreases with undercooling. From the change trend, *V/R* may be inversely proportional to undercooling, accordingly, it needs to be discussed.

## Discussions

**Figure 5:**
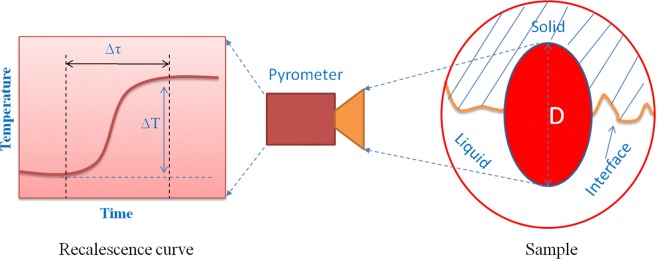
The schematic of HSC and pyrometer.

Generally, using pyrometer to measure the thermal history of solidification, the sample size is larger than the focus region of pyrometer. To illustrate the relation of recalescence rate and the interface growth velocity, the schematic is shown in Fig. 5. Given the diameter of focus region as *D*, the time for the solidification interface across the region as Δτ, then the growth velocity (*V*) and recalescence rate(R) can be expressed as

1 $$V=\frac{S}{t}=\frac{D}{\Delta \tau }$$

2 $$R=\frac{\varDelta T}{\varDelta \tau }$$

where *V* is the solidification interface growth velocity. Δ*T* is the recalescence degree or undercooling. Many experiments have shows that, the recalescence degree for primary phase solidification can be thought as linear with solidification undercooling, while that for pure metal or eutectic transformations, is nearly equal to the solidification undercooling^18^. in the present study, it is assumed that the Δ*T* equals to the undercooling. Then, it can be obtained,

3 $$\frac{V}{R}=\frac{D}{\varDelta T}$$

The growth velocity, *V* is determined by undercooling^19^; *D* is accurate by the distance between the pyrometer and the sample. Equation (3) indicates that, with the same undercooling, the larger value of *D*, the smaller value of recalescence rate *R* is. With a same distance between the pyrometer and the sample (constant *D*), the larger undercooling Δ*T* is, the larger value of recalescence rate *R* is.

If growth velocity *V* is calculated by theory model^19^, then the recalescence rate will be predicted according to Eq. (3), then more accurate cooling curves can be obtained combining with other model. Equation (3) also indicates that, using the same pyrometer, the recalescence rate in the cooling curve is settled by the distance and undercooling, which determined the focus region diameter *D* and interface growth velocity *V*, respectively.

For the present study, the focus region diameter *D* is about 3–6 mm on condition of the distance of 650 mm between sample and the pyrometer according to the equipment instruction. After substituting average value D = 4.5 mm in Eq. (3), the relation of *V/R* and Δ*T* can be calculated as shown in Fig. 4. It can be found that the experiment data trend is consistent with calculation, although there is small deviation. The deviation may be from: (1) Δ*T* of measurement is smaller than the accurate undercooling as it is from the recalescence degree; (2) the growth velocity is measured from the round sample surface but not smooth one, it is smaller than the accurate value.

Furthermore, it is often assumed that, *V* ∝ Δ*T*^*2*^ for the undercooled solidification^20^, thus from Eq. (3), *R* ∝ Δ*T*^*3*^, so we often see that the recalescence rate increases rapidly with undercooling.

As our knowledge, the recalescence degree is never higher than 0.2*T*_m_, although the largest undercooling can be larger than 0.2*T*_m_^21^. According to Eq. (3), the smallest *V/R* approach to *D*/0.2*T*_m_. For the current Fe-3.97 wt%B alloy, *T*_m_~1455 K, with *D* = 4.5 mm, so the extreme (smallest) value of *V/R* is 0.0155 mm/K. This result can be confirmed by the trend of experiment data in Fig. 4.

In addition, it is known that the critical radius of nucleation can be expressed as^22^

4 $${r}^{\ast }=\frac{2\gamma {T}_{m}}{\varDelta {H}_{f}\varDelta T}$$

Where γ (J m^−2^) is the interfacial tension; *T*_m_ is the melting point; Δ*H*_f_ is the latent heat of fusion (J m^−3^), Δ*T* is the undercooling (K). From Eqs. (3 and 4), gives

5 $$\frac{R{r}^{\ast }}{V}=k$$

where *k* is constant $(\frac{2\gamma {T}_{m}}{\varDelta {H}_{f}D})$. It indicates the critical radius, r* is inverse properties to the value of *R/V*, i.e. nucleation is connected with growth rate.

## Conclusions

The recalescence rate of cooling curves for rapid solidification have a similar trend with solidification interface growth velocity. Based on the experimental data of Fe-3.97 wt%B alloy, the theory relationship of recalescence rate and growth velocity is obtained as *V/R* = *D/*Δ*T*. It indicates that, the recalescence rate in the cooling curve does not only depends on the solidification undercooling, but also on the measured distance and measured area of pyrometer. With this relationship the interface growth velocity can be predicted by the cooling curves directly. Lastly, a relationship of the critical nucleation radius, recalescence rate and growth velocity is found as Rr^*^/V = constant, which suggests that the growth process has something to do with nucleation radius.

## Methods

To investigate the recalescence process in solidification, Fe-3.97 wt%B alloy sample is preferred. The sample weighed about 4 g was put in a quartz crucible covered by small amounts of B_2_O_3_ glass, then the crucible was placed around the medium positions of induction coils of a high frequency induction melting furnace. Then the sample were cyclically heated and cooled until the different undercoolings achieved. Thermal profiles of each sample were monitored by a one-color pyrometer with 10 ms delay time. An HSC instrumentation system with a high resolution of 1600 × 1600 pixels, I-Speed 221 was used to observe the recalescence process for each transformation. The instrument was focused on the sample surface from front view (the diameter of the crucible is 13.5 mm).

## Supplementary information

Supplementary Information

Supplementary Information 1

Supplementary Information 2

Supplementary Information 3

Supplementary Information 4

Supplementary Information 5

Supplementary Information 6
