# Supplementary material for: The recalescence rate of cooling curve for undercooled solidification
Source: Sci Rep. 2020 Jan 28;10:1380. doi: 10.1038/s41598-019-56079-6 (PMC6987091; doi:10.1038/s41598-019-56079-6)
Supplement: Supplementary file 1 — Supplementary Information [file 41598_2019_56079_MOESM1_ESM.doc]

**Supplementary of "The recalescence rate of cooling curve for undercooled solidification"**

Except for the manuscript sample Fe-3.97 wt% B, we also make the experiments for Fe-4.48wt% B, Fe-3.81wt%B, Ni-3.42wt%B and Ni-2.79wt%B alloys. The details are as follow:

1. **Fe-4.48wt% B alloy**

Fig.1 Cooling curves of hypoeutectic Fe-4.48wt%B alloy with different Δ*T*


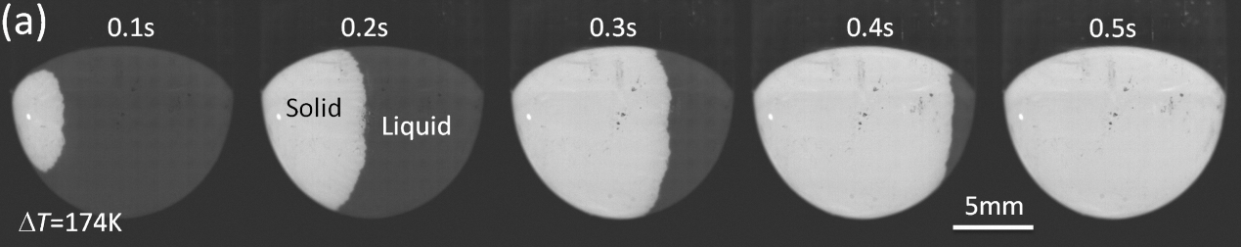


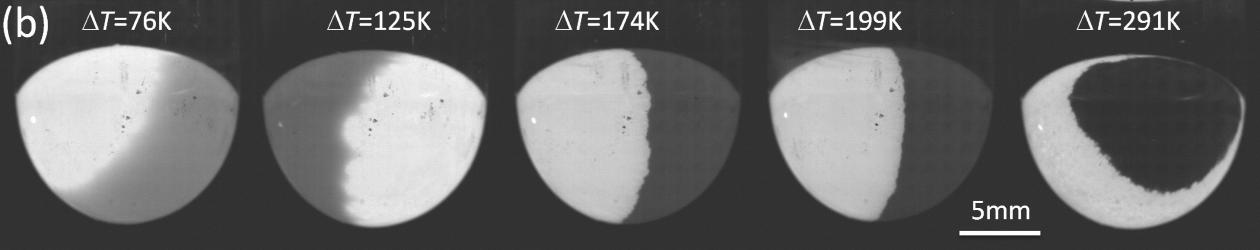


Fig. 2 High speed camera images: (a) solidification process of Δ*T*=174 K; (b) solidification interface with different Δ*T*

Fig. 3 Recalescence rate from cooling curves (a) and solidification interface growth velocities from high speed images (b)

Fig. 4 The ratio of recalescence rate and growth velocity

1. **Fe-3.81wt% Balloy**

Fig.1 Cooling curves of hypoeutectic Fe-3.81wt%B alloy


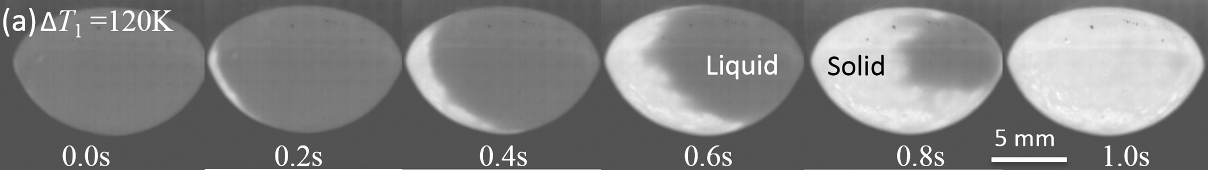


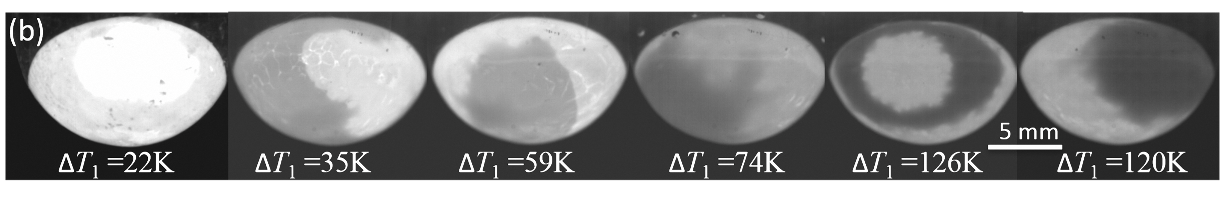


Fig. 2 HSC images: (a) solidification process of Δ*T*_1_=120 K; (b) solidification interface with different undercooling

Fig. 3 Recalescence rate from cooling curves (a) and solidification interface growth velocity from HSC images (b)

Fig. 4 The ratio of recalescence rate and growth velocity

1. **Ni-3.42wt%B alloy**


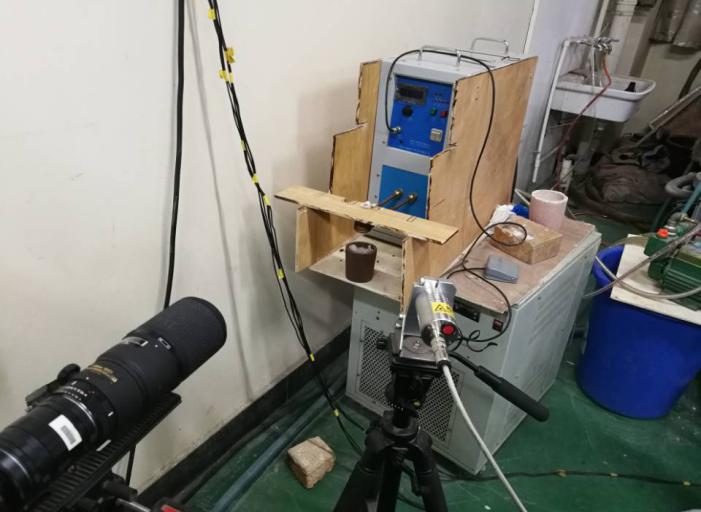

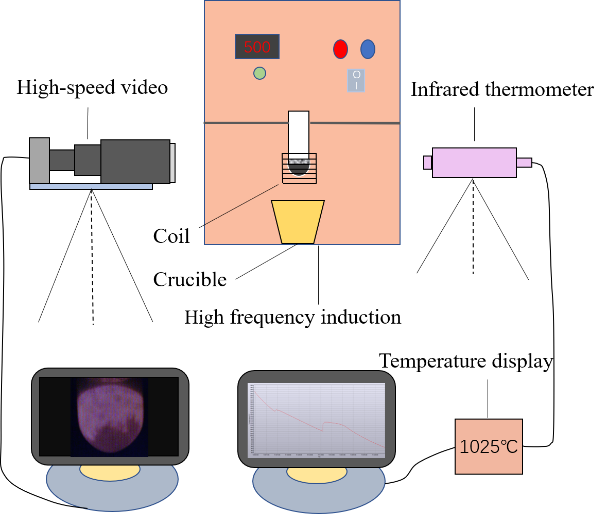


Fig. 1 Experimental installation and schematic diagram: (a) experimental installation; (b) experimental schematic

Fig. 2 Cooling curve of Ni-3.42wt%B


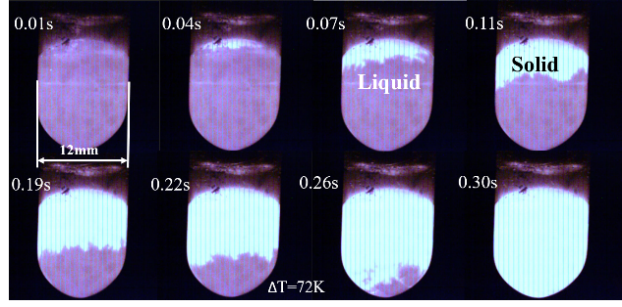


(a) The high-speed video images of Ni-3.42wt%B at undercooling of 72 K


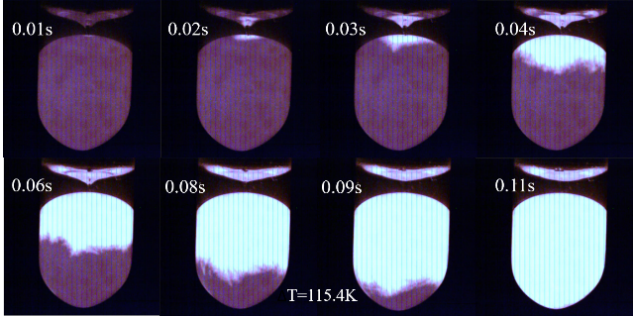


(b) The high-speed video images of Ni-3.42wt%B at undercooling of 115.4 K


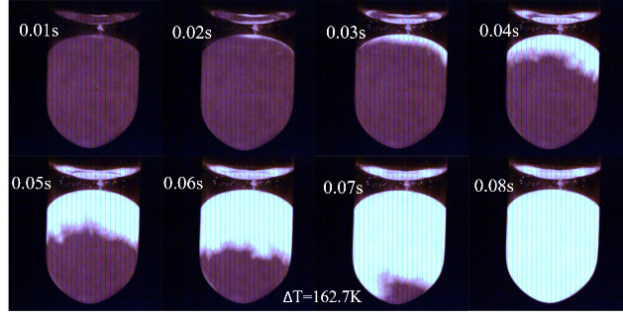


(c) The high-speed video images of Ni-3.42wt%B at undercooling of 162.7 K

Fig. 3 Interface morphology of Ni-3.42wt%B alloy at different time


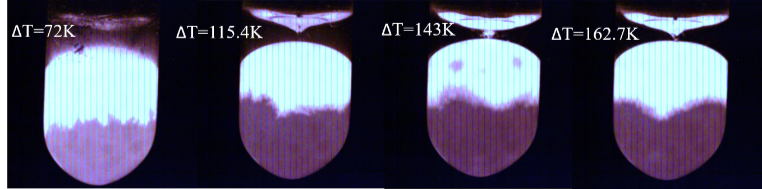


Fig. 4 Interface morphology of Ni-3.42wt%B alloy at different undercooling

The relationship between undercooling and growth rate of interface frontier is drawn in Fig. 5(a) by taking undercooling ∆*T* as independent variable and the growth rate of interface front as dependent variable from the growth rate measured in high-speed video.

Fig. 5(b) shows the relationship between interface movement distance and time of Ni-3.42wt%B alloy at undercooling of 49.8 K. It can be seen that the first half of the whole curve is basically a straight line, and the second half shows a rising trend of steps. The reason is that the growth rate of the precursor phase is much larger than that of the other phase in the eutectic transformation when the undercooling is large. At this time, the growth distance of the single phase is measured, so it is a straight line with almost invariable slope. In the later eutectic transformation, the rapid single-phase growth expelled a large number of excess solute atoms to the surrounding, which led to the acceleration of the growth of the other phase. The solute transversely diffused in front of the two phases, and the two phases grew in competition, which made the curve show a ladder-like upward trend.

Fig. 5(c) shows the relationship between the undercooling and the recalescence rate of Ni-3.42wt%B alloy.

(a)

(b)

(c)

Fig. 5 (a) Relationship of [undercooling](https://fanyi.baidu.com/#auto/auto/undercooling) and growth [rate](javascript:;); (b) Relationship of interface moving distance and time of Ni-2.79wt%B alloys at 49.8K; (c) Relationship of undercooling and recalescence rate

Fig. 6 shows the relationship between the ratio of the growth time of the interface front and the melt recalescence time with the undercooling. It can be seen that the ratio of growth time to melt recalescence time is fluctuates around 2.0, approximating a fixed value.

Fig. 6 The relation betweenthe ratio of growth time to recalescence time and undercooling

Fig. 7 The relation between the ratio of growth rate to recalescence rate and undercooling

**(4) Ni-2.79wt%B**

Fig.1 Cooling curve of Ni-2.79wt%B with different undercoolings


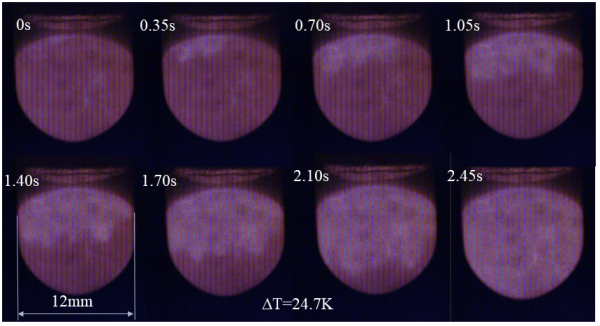


(a) The high-speed video images of Ni-2.79wt%B at undercooling of 24.7K


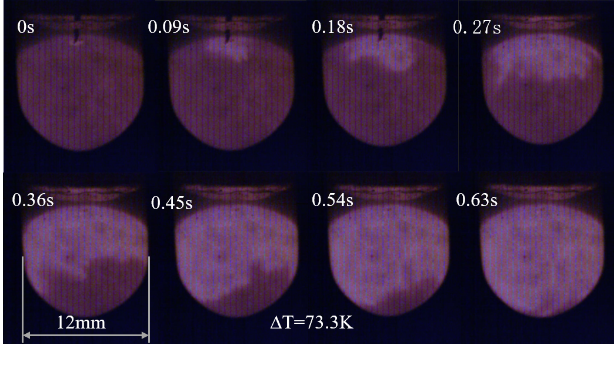


(b) The high-speed video images of Ni-2.79wt%B at undercooling of 73.3K


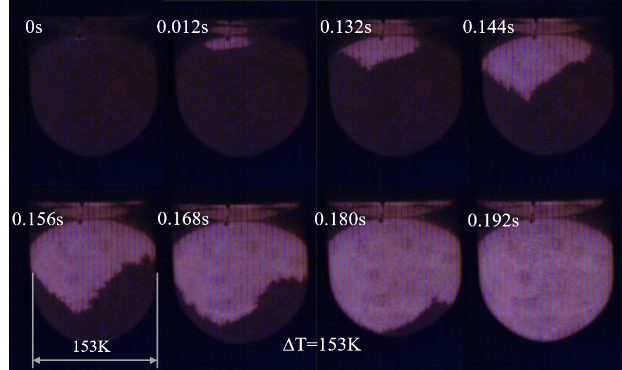


(c) The high-speed video images of Ni-2.79wt%B at undercooling of 153K

Fig.2 Interface morphology of Ni-2.79wt%B alloy at different time:

Fig.3 Relationship of [undercooling](https://fanyi.baidu.com/#auto/auto/undercooling) and growth [rate](javascript:;)

Fig.4 The relation between interface moving distance and time of Ni-2.79wt%B alloys at 153K

Fig.5 Relationship of [undercooling](https://fanyi.baidu.com/#auto/auto/undercooling) and recalescence rate

Fig.6 The relation between the ratio of growth time to recalescence time and undercooling

Fig.7 The relation betweenthe ratio of growth rate to recalescence rate and undercooling

All the result above confirm the theory relationship of recalescence rate and growth velocity as*V/R=D/*Δ*T.* All those results have been published anywhere.
